# Supplementary material for: A UMLS-based spell checker for natural language processing in vaccine safety
Source: BMC Med Inform Decis Mak. 2007 Feb 12;7:3. doi: 10.1186/1472-6947-7-3 (PMC1805499; doi:10.1186/1472-6947-7-3)
Supplement: Additional file 13 — RAP application directory. Sets up directory for RAP (RDF application for PHP) source code [file 1472-6947-7-3-S13.gz › rap/doc/phpdoc/__filesource/fsource_default__vocabularyOWL_C.php.html]

xml version="1.0" encoding="iso-8859-1"?


File Source for OWL\_C.php


# Source for file OWL\_C.php

Documentation is available at OWL\_C.php

```
1. <?PHP
2. // ----------------------------------------------------------------------------------
3. // OWL Vocabulary (Resource)
4. // ----------------------------------------------------------------------------------
5. // Version                   : 0.9
6. // Authors                   : Daniel Westphal (dawe@gmx.de)
7. //
8. // Description               : Wrapper, defining resources for all concepts of the Web
9. //                             Ontology Language (OWL). For details about OWL see:
10. //                             http://www.w3.org/TR/owl-ref/
11. //                                Using the wrapper allows you to define all aspects of
12. //                             the language in one spot, simplifing implementation and
13. //                             maintainence.
14. // ----------------------------------------------------------------------------------
15. // <BR><BR>History:<UL>
16. // <LI>06-25-2004             : $OWL_equivalentClass, $OWL_equivalentProperty, $OWL_Thing, $OWL_Nothing,
17. //                              $OWL_AllDifferent, $OWL_distinctMembers added (auer@informatik.uni-leipzig.de)</LI>
18. // <LI>05-24-2004              : $OWL_DeprecatedClass, $OWL_DeprecatedProperty, $OWL_priorVersion,
19. //                                $OWL_backwardCompatibleWith, $OWL_incompatibleWith (auer@informatik.uni-leipzig.de)</LI>
20. // <LI>03-26-2004              : $OWL:_AnnotationProperty and $OWL_DataRange added (auer@informatik.uni-leipzig.de)</LI>
21. // <LI>02-21-2003             : Initial version</LI>
22. // ----------------------------------------------------------------------------------


30. class OWL{

32. // OWL concepts

34. function ANNOTATION_PROPERTY()
35. {
36. return  new Resource(OWL_NS . 'AnnotationProperty');

38. }

40. function ALL_DIFFERENT()
41. {
42. return  new Resource(OWL_NS . 'AllDifferent');

44. }

46. function ALL_VALUES_FROM()
47. {
48. return  new Resource(OWL_NS . 'allValuesFrom');

50. }

52. function BACKWARD_COMPATIBLE_WITH()
53. {
54. return  new Resource(OWL_NS . 'backwardCompatibleWith');

56. }

58. function CARDINALITY()
59. {
60. return  new Resource(OWL_NS . 'cardinality');

62. }

64. function OWL_CLASS()
65. {
66. return  new Resource(OWL_NS . 'Class');

68. }

70. function COMPLEMENT_OF()
71. {
72. return  new Resource(OWL_NS . 'complementOf');

74. }

76. function DATATYPE()
77. {
78. return  new Resource(OWL_NS . 'Datatype');

80. }

82. function DATATYPE_PROPERTY()
83. {
84. return  new Resource(OWL_NS . 'DatatypeProperty');

86. }

88. function DATA_RANGE()
89. {
90. return  new Resource(OWL_NS . 'DataRange');

92. }

94. function DATATYPE_RESTRICTION()
95. {
96. return  new Resource(OWL_NS . 'DatatypeRestriction');

98. }

100. function DEPRECATED_CLASS()
101. {
102. return  new Resource(OWL_NS . 'DeprecatedClass');

104. }

106. function DEPRECATED_PROPERTY()
107. {
108. return  new Resource(OWL_NS . 'DeprecatedProperty');

110. }

112. function DISTINCT_MEMBERS()
113. {
114. return  new Resource(OWL_NS . 'distinctMembers');

116. }

118. function DIFFERENT_FROM()
119. {
120. return  new Resource(OWL_NS . 'differentFrom');

122. }

124. function DISJOINT_WITH()
125. {
126. return  new Resource(OWL_NS . 'disjointWith');

128. }

130. function EQUIVALENT_CLASS()
131. {
132. return  new Resource(OWL_NS . 'equivalentClass');

134. }

136. function EQUIVALENT_PROPERTY()
137. {
138. return  new Resource(OWL_NS . 'equivalentProperty');

140. }

142. function FUNCTIONAL_PROPERTY()
143. {
144. return  new Resource(OWL_NS . 'FunctionalProperty');

146. }

148. function HAS_VALUE()
149. {
150. return  new Resource(OWL_NS . 'hasValue');

152. }

154. function INCOMPATIBLE_WITH()
155. {
156. return  new Resource(OWL_NS . 'incompatibleWith');

158. }

160. function IMPORTS()
161. {
162. return  new Resource(OWL_NS . 'imports');

164. }

166. function INTERSECTION_OF()
167. {
168. return  new Resource(OWL_NS . 'intersectionOf');

170. }

172. function INVERSE_FUNCTIONAL_PROPERTY()
173. {
174. return  new Resource(OWL_NS . 'InverseFunctionalProperty');

176. }

178. function INVERSE_OF()
179. {
180. return  new Resource(OWL_NS . 'inverseOf');

182. }

184. function MAX_CARDINALITY()
185. {
186. return  new Resource(OWL_NS . 'maxCardinality');

188. }

190. function MIN_CARDINALITY()
191. {
192. return  new Resource(OWL_NS . 'minCardinality');

194. }

196. function NOTHING()
197. {
198. return  new Resource(OWL_NS . 'Nothing');

200. }

202. function OBJECT_CLASS()
203. {
204. return  new Resource(OWL_NS . 'ObjectClass');

206. }

208. function OBJECT_PROPERTY()
209. {
210. return  new Resource(OWL_NS . 'ObjectProperty');

212. }

214. function OBJECT_RESTRICTION()
215. {
216. return  new Resource(OWL_NS . 'ObjectRestriction');

218. }

220. function ONE_OF()
221. {
222. return  new Resource(OWL_NS . 'oneOf');

224. }

226. function ON_PROPERTY()
227. {
228. return  new Resource(OWL_NS . 'onProperty');

230. }

232. function ONTOLOGY()
233. {
234. return  new Resource(OWL_NS . 'Ontology');

236. }

238. function PRIOR_VERSION()
239. {
240. return  new Resource(OWL_NS . 'priorVersion');

242. }

244. function PROPERTY()
245. {
246. return  new Resource(OWL_NS . 'Property');

248. }

250. function RESTRICTION()
251. {
252. return  new Resource(OWL_NS . 'Restriction');

254. }

256. function SAME_AS()
257. {
258. return  new Resource(OWL_NS . 'sameAs');

260. }

262. function SAME_CLASS_AS()
263. {
264. return  new Resource(OWL_NS . 'sameClassAs');

266. }

268. function SAME_INDIVIDUAL_AS()
269. {
270. return  new Resource(OWL_NS . 'sameIndividualAs');

272. }

274. function SAME_PROPERTY_AS()
275. {
276. return  new Resource(OWL_NS . 'samePropertyAs');

278. }

280. function SOME_VALUES_FROM()
281. {
282. return  new Resource(OWL_NS . 'someValuesFrom');

284. }

286. function SYMMETRIC_PROPERTY()
287. {
288. return  new Resource(OWL_NS . 'SymmetricProperty');

290. }

292. function THING()
293. {
294. return  new Resource(OWL_NS . 'Thing');

296. }

298. function TRANSITIVE_PROPERTY()
299. {
300. return  new Resource(OWL_NS . 'TransitiveProperty');

302. }

304. function UNION_OF()
305. {
306. return  new Resource(OWL_NS . 'unionOf');

308. }

310. function VERSION_INFO()
311. {
312. return  new Resource(OWL_NS . 'versionInfo');
313. }

315. }


318. ?>
```

Documentation generated on Fri, 17 Dec 2004 16:16:45 +0100 by phpDocumentor 1.3.0RC3
